# Proportion and related factors of depression and anxiety for patients with pulmonary nodules in China: a outpatient-based cross-sectional study

**DOI:** 10.1038/s41598-025-17911-4

**Published:** 2025-08-31

**Authors:** Kangdi Cao, Zhuo Wang, Tong Zhou, Xinyan Wang, Zichen Wang, Lanxin Zhang, Bingjie Fan, Jinglei Li, Jiawei Wang, Xueqian Wang, Wei Hou

**Affiliations:** 1 Department of Graduate School, Beijing University of Chinese Medicine, Beijing, China; 2 Department of Oncology, Guang’anmen Hospital, China Academy of Chinese Medical Sciences, Beijing, China; 3 China Aerospace Science & Industry Corporation 731 Hospital, Beijing, China

**Keywords:** Pulmonary nodules, Depression, Anxiety, Related factors, History of occupational exposure, Insomnia, Lung cancer, Psychology, Cancer, Respiratory tract diseases

## Abstract

Investigating the proportion of depression and anxiety in patients with pulmonary nodules (PNs) and exploring the factors influencing them may help to optimize PNs management protocols and guide clinical decision-making. This study employed a cross-sectional design and included 260 patients attending the hospital’s outpatient clinic. The assessment of depression and anxiety in patients with PNs was conducted using the Hamilton Depression Scale (HAMD) and the Hamilton Anxiety Scale (HAMA), respectively. Demographic and clinical data were also collected. The factors influencing depression and anxiety in patients with PNs were analyzed using univariate analysis and binary logistic regression. The proportion of depression and anxiety in patients with PNs was 23.46% and 31.54%, respectively. The results of the binary logistic regression analysis indicated that insomnia (odds ratio [OR] = 7.343, 95%CI: 3.231–16.687) and loss of appetite (OR = 3.432, 95%CI: 1.474–7.991) were identified as independent risk factors for depression. The history of occupational exposure (OR = 4.154, 95%CI: 1.089–15.844), insomnia (OR = 4.162, 95% CI: 2.119–8.175), loss of appetite (OR = 2.326, 95%CI: 1.013–5.341), and chest tightness (OR = 3.155, 95% CI: 1.392–7.149) were significant independent predictors of anxiety. This study demonstrates that depression and anxiety are common with PNs in traditional Chinese medicine hospital. Insomnia and loss of appetite are independent risk factors for depression. History of occupational exposure, insomnia, loss of appetite, and chest tightness are independent risk factors for anxiety. Therefore, medical workers should pay close attention to the mood changes of these high-risk patients.

**Supplementary Information:**

The online version contains supplementary material available at 10.1038/s41598-025-17911-4.

## Introduction

Pulmonary nodules (PNs) are defined as hyperdense solid or subsolid lung shadows on imaging, with a diameter of ≤ 3 cm and a focal, round-like appearance. PNs may be isolated or multiple and are not accompanied by pulmonary atelectasis, hilar lymph node enlargement, or pleural effusion^1^. In recent years, there has been a notable rise in the detection rate of PNs due to heightened healthcare awareness and the growing popularity of low-dose computed tomography (LDCT) screening. This trend has given rise to considerable concern^2^.

The pathological diagnosis of PNs includes lung cancer. However, most PNs are benign, whether identified through screening or incidentally^3–5^. In the majority of cases, medical practitioners advise patients to undergo regular follow-up with LDCT. A PET-CT scan, biopsy, or surgical resection is undertaken only if the PNs have a malignant tendency^1^. However, the possibility of PNs progression during follow-up is perceived by patients as a “near-cancer” experience^6–8^. The experience has been demonstrated to cause significant psychological harm to the patient, which in turn has been shown to have a marked effect on the quality of life. Among the psychological burdens commonly experienced are depression and anxiety^9^;^10^. Moreover, due to a lack of understanding of PNs, patients’ inaccurate self-diagnosis often precedes professional assessment, which may also lead to depression and anxiety^11–13^. Research confirms that patients with depression and anxiety are frequently treated more aggressively than other patients, including high-frequency CT scans^14^. This phenomenon not only increases the burden on the healthcare system and results in a waste of medical resources, but also leads to increased radiation exposure and the risk of cancer^15,16^. At the same time, repeated examinations can intensify the patient’s state of depression and anxiety^17^. It is worth noting that long-term depression and anxiety can inhibit the immune function of patients^18–20^. Multiple meta-analyses further revealed that it was significantly associated with increased risk of lung cancer, worse prognosis, and increased mortality^15^;^16^. Therefore, research on depression and anxiety in PNs is of great importance to optimize PNs management plans and guide clinical decision-making. However, there is currently limited research on depression and anxiety in PNs. This study aims to assess the prevalence of depression and anxiety among PNs attending traditional Chinese medicine (TCM) hospital outpatient clinics using an internationally recognized scale and to determine the risk factors associated with depression and anxiety by collecting multidimensional information.

## Methods

### Participants

This study employed a cross-sectional design. Participants were recruited from the outpatient clinic of Guang’anmen hospital between 1 January and 31 December 2023. All participants provided informed consent before involvement in the study. Regarding the diagnostic criteria for PNs, we referred to the “Chinese expert consensus on the diagnosis and treatment of pulmonary nodules (2018 version)”^1^. The classification of hazard levels is based on the “China National Guideline of Classification, Diagnosis and Treatment for Lung Nodules (2016 Version)”^21^. The inclusion criteria were as follows: (1) the presence of PNs diagnosed by imaging, (2) the age range of 18 to 85 years, and (3) the ability to complete the questionnaire. The exclusion criteria were as follows: (1) diagnosis or history of malignant disease, (2) diagnosis or history of psychiatric disorders, or (3) an inability to comprehend the informed consent form and a refusal to sign it.

### Measurements

The proposal was approved by the Ethics Committee of Guang’anmen Hospital, China Academy of Chinese Medical Sciences (reference number: 2021-072-KY). The study was conducted in compliance with the Declaration of Helsinki. All patients used standardized assessment procedures. Potential participants were invited to participate in the study at the outpatient clinic. After the patients’ consent, the research group and the psychological evaluators interviewed the patients using a structured questionnaire. Firstly, the inclusion criteria and exclusion criteria for patients were determined. Secondly, the method and purpose of the study were explained to patients. Finally, after patients signed the informed consent, their information was collected. Demographic data and clinical data were collected by the research group. Our research group is composed of medical staff. They received systematic training before the study began. All depression and anxiety assessments were completed by psychological evaluators who did not know the study design and received standardized training. In the imaging information section, the research group will collect CT films of patients. Two experienced oncologists (Attending physician or associate chief physician) will evaluate the imaging information required for the study based on the same criteria using film. If the assessment results are inconsistent, a consensus will be reached through discussion with the third doctor (chief physician).

The data collected was divided into three sections. The first section comprised the patient’s demographic and clinical data, including name, gender, age, smoking history, history of occupational exposure (chemical exposure, radiation exposure, toxic exposure, etc.), family history of malignant tumor, body mass index (BMI), imaging data (included the number of PNs, the size of PNs, and the presence of malignant signs). Malignant signs in the imaging presentation of PNs included one or more of the following: lobulation, irregular morphology, burr sign, vacuole sign, pleural depression sign, and vascular cluster sign^1^. These variables have been employed in previous studies^22^;^23^. The relationship between these factors and depression/anxiety remains the subject of ongoing research, with no consensus yet reached among the academic community. Further analysis is required to elucidate the complex interplay between these variables. To analyse the correlation between depression, anxiety and clinical symptoms, six common clinical symptoms were added to the study, including cough, chest tightness, shortness of breath, insomnia, palpitation, and loss of appetite^24^;^25^. The clinical symptoms were assessed using the “Guidelines for Clinical Research of Traditional Chinese Drug Research”^26^.

In the second part, the Hamilton Depression Scale (HAMD) was employed to assess the depression state of the patients^27^. HAMD was a 17-item scale. The severity of depression was gauged by the frequency and intensity of the patient’s symptoms over the past week. Each item was classified into one of five levels or three levels, with 0 to 4 points assigned to five levels and 0 to 2 points assigned to three levels, respectively. The scores for each item were aggregated to obtain the total score for the scale, which was indicative of the severity of depression. The scale was divided as follows: no depression (0–7); mild depression (8–16); moderate depression (17–23); and severe depression (≥ 24)^28^.

In the third part of the study, the anxiety state of the patients was evaluated using the Hamilton Anxiety Scale (HAMA)^29^. HAMA was a 14-item scale. The severity of anxiety was gauged by the frequency and intensity of the patient’s symptoms over the past week. Each item was classified into one of five levels, with a score ranging from 0 to 4. The scores for each item were summed to obtain the total score for the scale. The total score was indicative of the severity of anxiety. The scale was divided as follows: no/minimal anxiety (0–7); mild anxiety (8–14); moderate (15–23); severe (≥ 24)^30^.

The selection of HAMD and HAMA was based on the following reasons: (1) HAMD and HAMA are authoritative scales for assessing depression and anxiety and are widely used in clinical practice^31^;^32^. (2) HAMD and HAMA are scales used by professionals, which can reduce subjective bias^33^.

### Statistical analyses

The statistical analyses were conducted using the IBM SPSS Statistics 26.0 software. The patient’s demographic and clinical data were subjected to descriptive analysis. The counting data were described as frequencies and percentages. The normally distributed quantitative data were described as mean and standard deviation (‾*X* ± *S*) and the others by median and associated interquartile range (M (P25, P75)). In univariate analysis, the counting data were analyzed using either the chi-square test or Fisher’s exact probability method. The normally distributed quantitative data were analyzed using the t-test and the others using the Mann–Whitney U test. The multivariate analysis was conducted using binary logistic regression. The objective was to assess independent associations regarding depression and anxiety. P value < 0.05 was considered statistically significant.

## Result

### Characteristics of all participants

A total of 269 patients agreed to participate in this study. 9 patients were excluded due to incomplete data. A total of 260 patients completed informed consent and information collection, with a response rate of 96.7%. Based on a screening study of depression in patients with PNs (depression accounted for 19.4%), a sample size of 255 cases was calculated using PASS 15 software at α = 0.05 and an allowable error of 5%. Considering the 5% invalid questionnaire, at least 269 patients were included. Therefore, the sample size of this study meets the requirements.

Among the 260 survey responses, 66.15% were female participants and the rest were male (33.85%). The age of patients was mainly middle-aged and elderly (83.46%). The average BMI was 24.17 ± 2.95. In addition, 13.85% of the patients smoked, 5.77% of the patients had a history of occupational exposure, and 36.54% of the patients had a family history of malignant tumour. In terms of imaging information, the classification of hazard levels of patients included was mainly medium risk (69.23%). Table 1 describes the characteristics of the included patients.

**Table 1:** Characteristics of all participants.

Variable		Frequency (*N* = 260)	Percentage (%)	Mean (SD)	Range
Age (years)	≤ 44	43	16.54		
	45 ~ 64	150	57.69		
	≥ 65	67	25.77		
Gender	Male	88	33.85		
	Female	172	66.15		
Smoking history	Yes	36	13.85		
	No	224	86.15		
History of occupational exposure	Yes	15	5.77		
	No	245	94.23		
Family history of malignant tumour	Yes	95	36.54		
	No	165	63.46		
The number of PNs	Single	42	16.15		
	Multiple	218	83.85		
The size of PNs	<8 mm	149	57.31		
	≥ 8 mm	111	42.69		
Malignant signs	Yes	34	13.08		
	No	226	86.92		
Classification of hazard levels	Low risk	37	14.23		
	Medium risk	180	69.23		
	High risk	43	16.54		
BMI				24.17(2.95)	16.22–33.87
Cough	Yes	98	37.69		
	No	162	62.31		
Chest tightness	Yes	85	32.69		
	No	175	67.31		
Shortness of breath	Yes	110	42.31		
	No	150	57.69		
Insomnia	Yes	132	50.77		
	No	128	49.23		
Palpitation	Yes	92	35.38		
	No	168	64.62		
Loss of appetite	Yes	41	15.77		
	No	219	84.23		

### Proportion of depression and anxiety of PNs

**Fig. 1:**
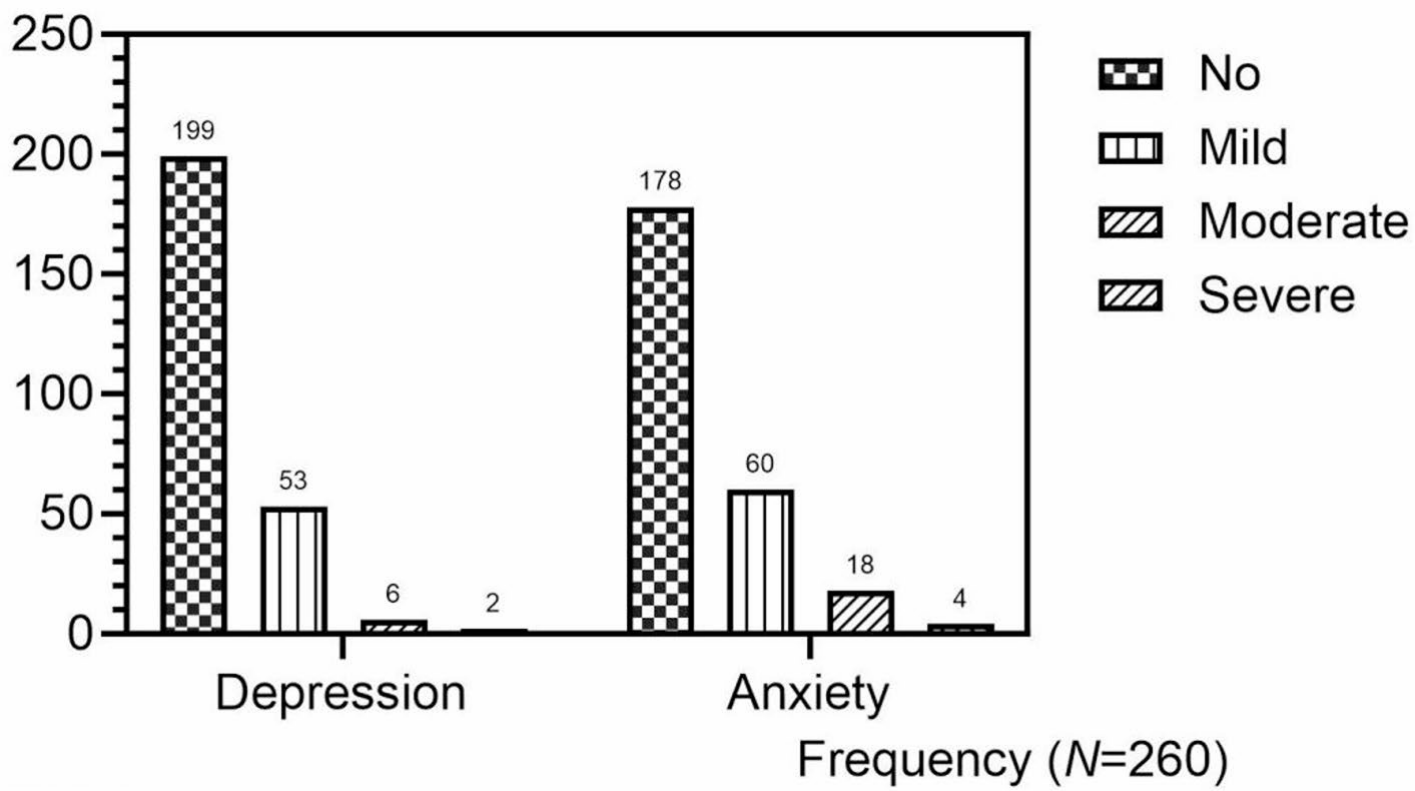
The distribution of depression and anxiety in patients with PNs.

Of the 260 patients with PNs, 53 (20.38%) had mild depression, 6 (2.31%) had moderate depression, and 2 (0.78%) had severe depression. Therefore, a total of 61 patients (23.46%) were considered to have depression. There were 60 (23.08%) classified as mild anxiety, 18 (6.92%) classified as moderate anxiety, and 4 (1.54%) classified as severe anxiety. So, a total of 82 patients (31.54%) were identified as anxiety. (Fig. 1)

### Univariate analysis of factors in depression and anxiety

The univariate analysis results of depression showed significant differences in variables such as cough (*χ*^*2*^ *=* 5.848, *P* = 0.016), chest tightness (*χ*^*2*^ *=* 31.740, *P* < 0.001), shortness of breath (*χ*^*2*^ *=* 29.042, *P* < 0.001), insomnia (*χ*^*2*^ *=* 37.902, *P* < 0.001), palpitation (*χ*^*2*^ *=* 25.243, *P* < 0.001), and loss of appetite (*χ*^*2*^ *=* 17.377, *P* < 0.001). (Table 2) The results of the univariate analysis for anxiety demonstrated significant differences in several variables, including the history of occupational exposure (*χ*^*2*^ *=* 5.972, *P* = 0.015), cough (*χ*^*2*^ *=* 7.725, *P* = 0.005), chest tightness (*χ*^*2*^ *=* 51.377, *P* < 0.001), shortness of breath (*χ*^*2*^ *=* 39.646, *P* < 0.001), insomnia (*χ*^*2*^ *=* 29.570, *P* < 0.001), palpitation (*χ*^*2*^ *=* 34.308, *P* < 0.001), and loss of appetite (*χ*^*2*^ *=* 11.031, *P* = 0.001). (Table 3) There were no significant differences in the distribution of depression and anxiety among gender, age, BMI, smoking history, family history of malignant tumour, size of PNs, number of PNs, malignant signs, and classification of hazard levels. (Tables S1, S2)

**Table 2:** Univariate analysis of factors in depression.

Variable		Depression	Non-depression	χ^2^	*P*
Cough				5.848	0.016
	Yes	31	67		
	No	30	132		
Chest tightness				31.740	<0.001
	Yes	38	47		
	No	23	152		
Shortness of breath				29.042	<0.001
	Yes	44	66		
	No	17	133		
Insomnia				37.902	<0.001
	Yes	52	80		
	No	9	119		
Palpitation				25.243	<0.001
	Yes	38	54		
	No	23	145		
Loss of appetite				17.377	<0.001
	Yes	20	21		
	No	41	178		

**Table 3:** Univariate analysis of factors in anxiety.

Variable		Anxiety	Non-anxiety	χ^2^/t	*P*
History of occupational exposure				5.972	0.015
	Yes	9	6		
	No	73	172		
Cough				7.725	0.005
	Yes	41	57		
	No	41	121		
Chest tightness				51.377	<0.001
	Yes	52	33		
	No	30	145		
Shortness of breath				39.646	<0.001
	Yes	58	52		
	No	24	126		
Insomnia				29.570	<0.001
	Yes	62	70		
	No	20	108		
Palpitation				34.308	<0.001
	Yes	50	42		
	No	32	136		
Loss of appetite				11.031	0.001
	Yes	22	19		
	No	60	159		

### Multivariate analysis of factors in depression and anxiety

**Fig. 2:**
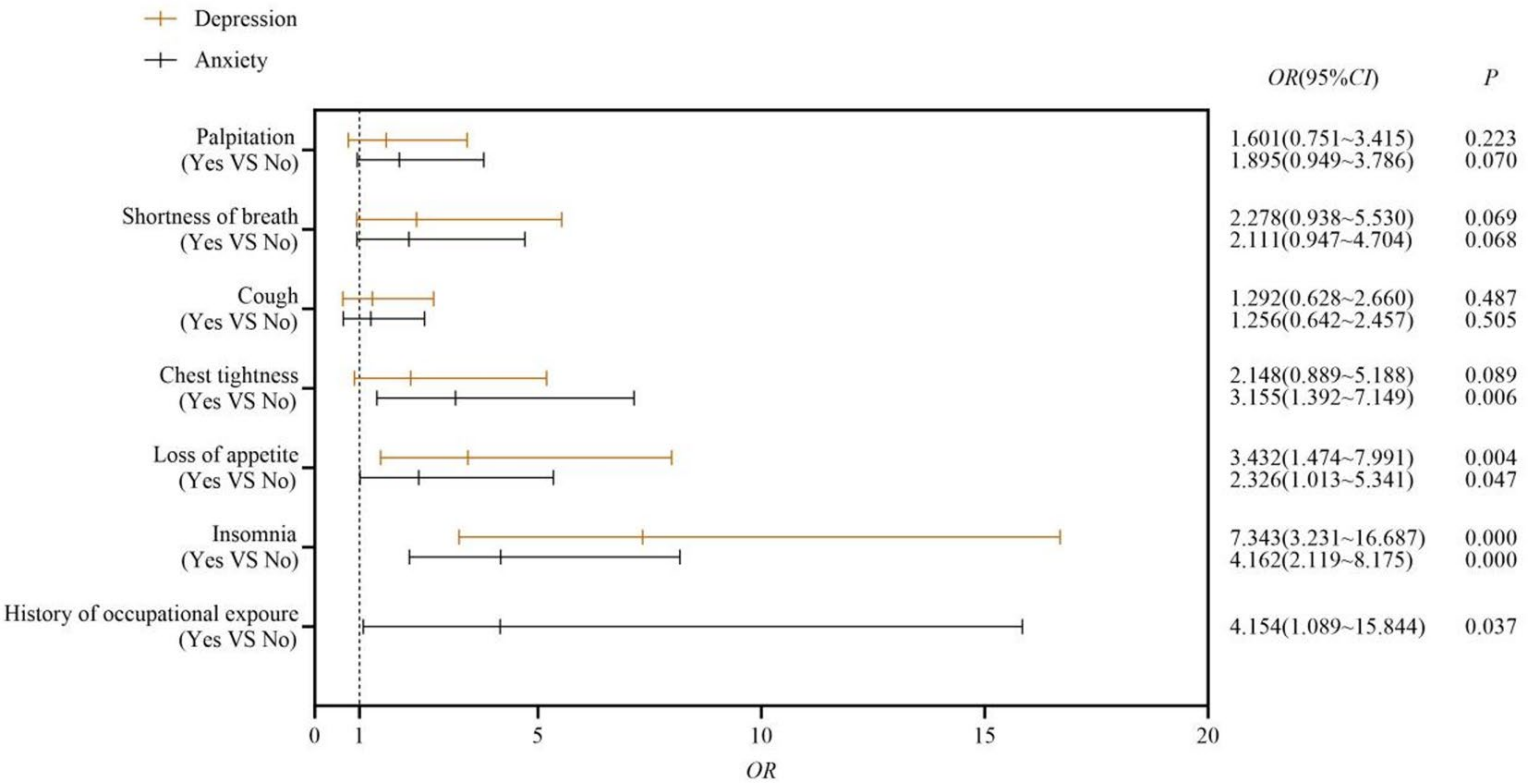
Multivariate analysis of factors in depression and anxiety.

Following the univariate analyses, binary logistic regression analyses were performed on the screened variables. Figure 2 illustrates the significant related factors of depression and anxiety. The results showed that insomnia (odds ratio [OR] = 7.343, 95%CI: 3.231–16.687) and loss of appetite (OR = 3.432, 95%CI: 1.474–7.991) were identified as independent risk factors for depression. The history of occupational exposure (OR = 4.154, 95%CI: 1.089–15.844), insomnia (OR = 4.162, 95% CI: 2.119–8.175), loss of appetite (OR = 2.326, 95%CI: 1.013–5.341), and chest tightness (OR = 3.155, 95% CI: 1.392–7.149) were significant independent predictors of anxiety.

## Discussion

The aim of this study was to measure the proportion of depression and anxiety in outpatients with PNs and to explore the related factors associated with depression and anxiety. We recruited patients with PNs who were first seen at an outpatient clinic in 2023. It was found that the proportion of depression and anxiety accounted for 23.46% and 31.54%, respectively.

Regarding the prevalence of depression and anxiety, Xiao R et al. employed the Hospital Anxiety and Depression Scale (HADS) to assess psychological distress in a cohort of 334 patients with PNs. Their findings revealed that 14.67% of the patients screened positive for depression and 17.96% for anxiety^14^. Zhuang W et al. employed the HADS to identify instances of depression and anxiety among patients with PNs. The results demonstrated that 1185 patients with PNs displayed depression in 27.0% of cases and anxiety in 42.1% of cases^9^. The results obtained by Li L et al. using the HADS fell within the middle range, with depression and anxiety accounting for 19.4% and 31.8% respectively^34^. Furthermore, some studies utilized the Patient Health Questionnaire-9 (PHQ-9) and the Generalised Anxiety Disorder-7 (GAD-7) to screen for depression and anxiety in patients with PNs. The results demonstrated a prevalence of 29.73% for depression and 28.86% for anxiety^35^. The study was conducted primarily in the outpatient clinics of TCM hospitals. HAMD and HAMA were employed in our investigation. HAMD represented the gold standard for rating depression^31^;^36^whereas HAMA was one of the most frequently utilized instruments for assessing anxiety in clinical settings^32^. This represents the authority and universality of HAMA and HAMD. Specifically, the HAMD and HAMA are rating scales used by professionals and not filled out by patients themselves^33^. Compared to other self-rating scales, it can reduce subjective bias. Meanwhile, HAMD and HAMA involve multiple dimensions of assessing anxiety and depression, such as emotions, somatization, sleep, cognition, with broad coverage and comprehensive symptom assessment. Therefore, the accuracy and sensitivity of HAMD and HAMA are reliable. The results were comparable to those previously researched. This indicated that depression and anxiety were relatively prevalent among patients with PNs, representing a significant challenge for this patient population. Therefore, it is imperative to acknowledge the significance of mental health in patients afflicted with PNs. Early identification and timely psychological interventions are of considerable benefit to these patients. This also suggests that it is necessary to consider the mental health status of patients with PNs when optimizing management plans and formulating healthcare policies in the future.

Regarding potential risk factors for depression and anxiety in patients with PNs, our findings indicated that a history of occupational exposure was a significant risk factor for anxiety in patients with PNs. This may be related to the fact that the occupational exposure population is in a potentially carcinogenic environment for a long time and has a higher sensitivity to the risk of malignant progression of abnormal lung lesions. Our statistical occupational exposure factors included chemical exposure, radiation exposure, toxic exposure, and so on. Previous studies have shown that these factors may increase the risk of cancer^37–40^. Compared with the general population, these patients tend to have a more direct understanding of the correlation between occupational exposure factors and tumor occurrence^41^. Their anxiety not only stems from the worry about the nature of the current PNs, but also includes the fear that previous exposure history may lead to disease progression, as well as the worry about the treatment prognosis. This superimposed psychological burden significantly increases the possibility of anxiety. There were no significant differences in depression and anxiety by gender, age, or family history of the tumour. This finding was consistent with the results of the study conducted by Li et al.^34^. However, Zhuang et al. demonstrated that younger individuals and patients with a family history of tumors exhibited elevated levels of anxiety^9^while Xiao et al. indicated that females were more likely to have anxiety than males^14^. Compared with these two studies, our study was conducted in the hospital of TCM. Sample size, regional deviation, and cultural background may be important reasons for the differences in results. In terms of sample size, females accounted for 66.15%, while young people accounted for a lower percentage. Uneven sample sizes and small overall sample sizes may reduce the efficacy of statistical tests^42^. In the geographical and TCM cultural environment of the study population, young people and women may have a more positive understanding of the clinical outcome of PNs (such as agreeing with the concept of “preventive treatment of disease” in TCM). And patients with a family history of tumor may form stronger psychological resilience due to long-term family care experience. These weaken the association of the above factors with emotional disorders. This also suggests that the combination of TCM and modern medicine may bring more benefits to patients with PNs. Furthermore, Zhuang et al. demonstrated that the number of PNs was a significant positive predictor of depression and anxiety^9^. However, the results of our study did not indicate a correlation between imaging manifestations (including PNs size and number), classification of hazard levels and depression and anxiety. We speculate that this is related to the distribution characteristics of PNs in the sample. For example, the high proportion of multiple pulmonary nodules (83.85%) and non-malignant signs (86.92%) may reduce the efficiency of the statistical test^42^. It also reflects the difference in attention to imaging indicators between different study populations. The study population may pay more attention to the doctor’s verbal assessment of PNs risk, rather than purely numerical values of number or diameter. This also explains why imaging findings are not associated with depression and anxiety, suggesting that the psychological state of patients is more directly driven by subjective cognition than objective indicators. Therefore, doctors’ adequate education and communication to patients with PNs in the process of diagnosis and treatment may be an important measure to reduce depression and anxiety.

The presence of clinical symptoms is typically the primary motivation for patients to seek medical attention. The extant literature indicated that subjective symptoms were closely associated with psychological states. It is possible that clinical symptoms may exacerbate depression and anxiety, and conversely, depression and anxiety may precipitate the onset of clinical symptoms^43^;^44^. Our results showed that insomnia and loss of appetite were independent factors that contributed to depression and anxiety. Prior research has demonstrated that insomnia increases the risk of depression and anxiety^45,46^. The regulation of insomnia has been demonstrated to result in the alleviation of depression and anxiety^47,48^. These findings suggested that insomnia was a key risk factor for depression and anxiety. In addition to insomnia, symptoms of the digestive system were one of the symptom clusters that depression and anxiety patients suffered^49^. Studies have shown that symptoms of the digestive system were more common in patients with depression and anxiety than in patients without depression and anxiety^50^. Our research revealed that loss of appetite was an independent influencing factor for depression and anxiety in symptoms of the digestive system, which was consistent with previous studies^51–53^. Furthermore, we found that chest tightness was an independent risk factor for depression. The extant literature indicated that respiratory symptoms exerted an independent influence on the development of anxiety^9^;^43^. However, specific symptoms had not been statistically analyzed, which also supported the results of the present study. Overall, these results suggested that we need to pay attention to the mental health of patients with insomnia, chest tightness, and loss of appetite. It was recommended that improvements be made as soon as these symptoms manifest to prevent the development of psychological problems. This will help reduce the incidence of lung cancer. In terms of specific treatment, for insomnia, chest tightness, and loss of appetite, modern medicine can use cognitive behavior therapy, respiratory training guidance, and dietary adjustment, respectively^54–57^. Severe cases can be treated with corresponding medications. For patients in a TCM hospital, TCM decoction and acupuncture are also important measures to improve symptoms.

There are several limitations to our study, firstly the cross-sectional design employed did not allow us to ascertain the causal relationship between the variables and depression and anxiety. Furthermore, The sample size of the study was restricted by insufficient funding. A correlation analysis between symptom severity and depression/anxiety severity was not performed. Thirdly, due to financial and temporal constraints, only six common clinical symptoms in patients with PNs were selected for analysis, and a comprehensive and complete symptom analysis was not performed. Other potential symptoms may affect the research results. Finally, this study is a single-center study, and the included population is Chinese people in a TCM hospital, which may have some selection bias. This also limits the universal applicability of the results to a certain extent. However, the results of this study also have certain reference value for other Chinese people and East Asian countries with traditional medical backgrounds (such as South Korea and Japan). This suggests that in the face of people with depression and anxiety, the combination of traditional medicine and modern medicine can be used for education and intervention. In addition, the results of this study found that occupational exposure history was a risk factor for anxiety, which was consistent with modern medical theory. Therefore, the results also have some enlightenment for other races in the context of modern medicine. Due to the shortcomings of this study, it is necessary to further clarify the results of the study through a multicenter, large-sample design, including people of different races, medical systems, and cultural backgrounds. This will also help to more accurately define the applicable boundaries of various risk factors and enhance the clinical transformation value of research results. In addition, multicenter, large-sample longitudinal studies are also necessary in the future to clarify the causal relationship between depression, anxiety, and related factors, as well as the clinical efficacy of psychological interventions.

## Conclusion

Depression and anxiety were very common with PNs in TCM hospital. Insomnia and loss of appetite were independent risk factors for depression. The history of occupational exposure, insomnia, loss of appetite, and chest tightness were independent risk factors for anxiety. The results not only provide a target for clinical psychological intervention, but also provide a basis for the optimization of PNs management scheme and healthcare policy making. Medical workers should pay close attention to mood changes in these high-risk patients and help them alleviate their negative emotions. Clinical experts and managers should also consider the mental health of patients with PNs when making schemes and policies, so as to achieve the efficient use of medical resources and the overall improvement of the quality of life of patients.

## Supplementary Information

Below is the link to the electronic supplementary material.

Supplementary Material 1

## Data Availability

All data generated or analysed during this study are included in this published article.
