# Supplementary material for: Proportion and related factors of depression and anxiety for patients with pulmonary nodules in China: a outpatient-based cross-sectional study
Source: Sci Rep. 2025 Aug 31;15:32029. doi: 10.1038/s41598-025-17911-4 (PMC12399759; doi:10.1038/s41598-025-17911-4)
Supplement: Supplementary file 1 — Supplementary Material 1 [file 41598_2025_17911_MOESM1_ESM.docx]

Table S1 Analysis of depression influencing factors in patients with pulmonary nodules

| Variable | | Depression | Non-depression | *χ^2^*/*t* | *P* |
| --- | --- | --- | --- | --- | --- |
| Age (years) |  |  |  | 0.612 | 0.736 |
|  | ≤44 | 10 | 33 |  |  |
|  | 45 ~ 64 | 33 | 117 |  |  |
|  | ≥65 | 18 | 49 |  |  |
| Gender |  |  |  | 0.040 | 0.842 |
|  | Male | 20 | 68 |  |  |
|  | Female | 41 | 131 |  |  |
| Smoking history |  |  |  | 2.268 | 0.132 |
|  | Yes | 12 | 24 |  |  |
|  | No | 49 | 175 |  |  |
| History of occupational exposure |  |  |  | 0.864 | 0.353 |
|  | Yes | 5 | 10 |  |  |
|  | No | 56 | 189 |  |  |
| Family history of malignant tumour |  |  |  | 1.940 | 0.379 |
|  | Yes | 20 | 75 |  |  |
|  | No | 41 | 124 |  |  |
| The number of PNs |  |  |  | 0.543 | 0.461 |
|  | Single | 8 | 34 |  |  |
|  | Multiple | 53 | 165 |  |  |
| The size of PNs |  |  |  | 0.095 | 0.758 |
|  | ＜8mm | 36 | 113 |  |  |
|  | ≥8mm | 25 | 86 |  |  |
| Malignant signs |  |  |  | 0.180 | 0.672 |
|  | Yes | 7 | 27 |  |  |
|  | No | 54 | 172 |  |  |
| Classification of hazard levels |  |  |  | 3.634 | 0.163 |
|  | Low risk | 5 | 32 |  |  |
|  | Medium risk | 48 | 132 |  |  |
|  | High risk | 8 | 35 |  |  |
| BMI |  |  |  | 0.085 | 0.932 |

Note: T-test satisfies normal distribution and homogeneity of variance.

Table S2 Analysis of anxiety influencing factors in patients with pulmonary nodules

| Variable | | Anxiety | Non-anxiety | *χ^2^*/*t* | *P* |
| --- | --- | --- | --- | --- | --- |
| Age (years) |  |  |  | 1.626 | 0.443 |
|  | ≤44 | 15 | 28 |  |  |
|  | 45 ~ 64 | 50 | 100 |  |  |
|  | ≥65 | 17 | 50 |  |  |
| Gender |  |  |  | 1.798 | 0.180 |
|  | Male | 23 | 65 |  |  |
|  | Female | 59 | 113 |  |  |
| Smoking history |  |  |  | 2.831 | 0.092 |
|  | Yes | 7 | 29 |  |  |
|  | No | 75 | 149 |  |  |
| Family history of malignant tumour |  |  |  | 0.000 | 0.991 |
|  | Yes | 30 | 65 |  |  |
|  | No | 52 | 113 |  |  |
| The number of PNs |  |  |  | 0.204 | 0.651 |
|  | Single | 12 | 30 |  |  |
|  | Multiple | 70 | 148 |  |  |
| The size of PNs |  |  |  | 1.826 | 0.177 |
|  | ＜8mm | 52 | 97 |  |  |
|  | ≥8mm | 30 | 81 |  |  |
| Malignant signs |  |  |  | 0.256 | 0.613 |
|  | Yes | 12 | 22 |  |  |
|  | No | 70 | 156 |  |  |
| Classification of hazard levels |  |  |  | 2.036 | 0.361 |
|  | Low risk | 8 | 29 |  |  |
|  | Medium risk | 59 | 121 |  |  |
|  | High risk | 15 | 28 |  |  |
| BMI |  |  |  | -0.448 | 0.655 |

Note: T-test satisfies normal distribution and homogeneity of variance.
